# Application of dynamic accuracy evaluation for press systems based on orthogonal design method

**DOI:** 10.1016/j.mex.2024.102568

**Published:** 2024-01-12

**Authors:** Shunyao Wang

**Affiliations:** School of Mechanical and Electrical Engineering, Jinling Institute of Technology, Nanjing, Jiangsu 211169, China

**Keywords:** Dynamic accuracy evaluation, Ultra-precision mechanism, Coupling effects, Simplify analysis, Orthogonal sample analysis method of dynamic accuracy.

## Abstract

The variation of dynamic accuracy for press systems is the nonlinear phenomenon that results from the consideration of contact and impact on the deformation of transmission mechanism, usually revolute joint and translation joint. The influence is especially obvious in the ultra-precision mechanism, which can cause the vibration and unstabitily of position and machining accuracy would be failure. As usual, the dynamic accuracy is used to evaluate the ability of press systems, which is also the important design object. Due to the stronger nonlinear of dynamic accuracy, especially for the effect of coupling factors, the mathematical analysis method plays an important role in the study of dynamic behavior for press systems. This work proposes the new approach to conduct the simplified dynamic accuracy analysis based on the orthogonal design method, which optimize the reasonability of sample collection. The proposed method is compared with the traditional approach, which illustrates the advantage and efficiency for the dynamic accuracy analysis of press systems.

• Developed dynamic accuracy analysis is observed to be effective for the stability evaluation of press systems.
• The simplified model of coupling effect analysis is established based on orthogonal design method.
• No need to collect a large amount of data for comparison and the reliable nonlinear analysis is conducted with simplified model.

## Graphical abstract

**Figure:**
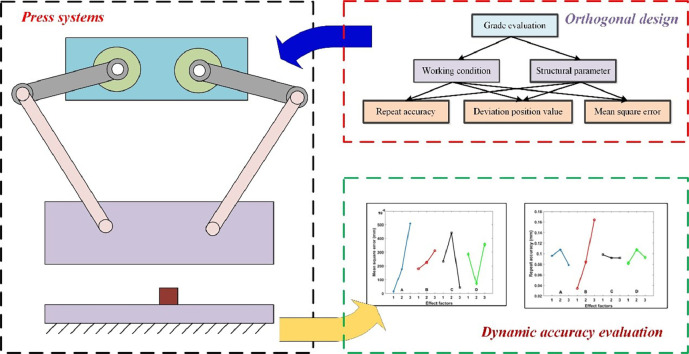

Specifications Table

**Table:** 

Subject area:	Engineering
More specific subject area:	Dynamic accuracy analysis of ultra-precision mechanism.
Method name:	Orthogonal sample analysis method of dynamic accuracy
Name and reference of original method:	N/A
Resource availability:	N/A

## Introduction

The press systems is one of main machine for manufacturing the ultra-precision industrial parts, and the dynamic accuracy is the important evaluation elements for the ability of press systems [1], [2], [3]. Many factors have direct effects for the variation of dynamic accuracy, which is also close related with the service life and process efficiency. Erkaya [[4], [5]] conducted the dynamic behavior test of high-speed mechanism, and the results showed that the clearance joint would cause the vibration of this mechanism. Considering the effects of clearance, the dynamic vibration characteristics of ball bearing was revealed and the transient response was illustrated by the acceleration. Xiao [6] built the kinematic accuracy analysis model of press systems, and the Monte Carlo method was used to evaluate the reliability of this mechanism. Based on the transmission mechanism of press systems, Zheng [7] analyzed the drift phenomenon of bottom dead position for slider, which represented the relationship between working condition and stability. In addition, Wu [8] proposed an comprehensive method to describe the dynamic behavior of ultra-precision mechanism, and the vibration response was performed by the experiment. Chen [9] developed a comparison study of dynamic characteristics analysis for press systems, and the mathematical statistics method was employed to illustrate the distribution of kinematic errors. Based on the stability analysis, the dynamic response of mechanism is sensitive to the change of clearance joint, working condition and structural parameter. For example, the increase of clearance size can be expanded the motion range of joint element, and the dynamic behavior of mechanism is also changed. Meanwhile, the higher working speed would enhance the contact-impact force of mechanical systems element. And then, the dynamic accuracy of mechanism may be invalidated. The traditional evaluation of dynamic accuracy for press systems is only obtained by the maximum deviation value of extreme position for slider, and the coupled analysis of design parameters affect on the dynamic accuracy is conducted based on the large amount data in the different conditions. Then, it is necessary to proposed an approach to simplify the influence of coupled parameters on the dynamic accuracy of ultra-precision mechanism.

The object of this work is to propose a new evaluation method of dynamic accuracy for press systems, which can be used to develop the parameter design of ultra-precision mechanism. The potential applications of this approach is illustrated by the engineering case. The introduction of orthogonal design method is simplified the design process of coupled factors effects and optimize calculation efficiency during the parameters design.

## Method details

### Comprehensive evaluation systems of dynamic accuracy

The dynamic accuracy of press systems is the deviation between theoretical value and real value at the extreme lowest position of slider, which is the transient value [10]. The repeat accuracy, the deviation position value and the mean square error of extreme lowest position are employed to evaluate the dynamic accuracy of press system. The repeat accuracy represents the maximum deviation between extreme lowest position and average value in the sampling interval, which can reflect the stamping ability of press systems and the effects of product accuracy. The expression can be given as:

(1) $$\gamma_{1}=max\left\{\left|y\left(i\right)-y_{a}\right|\right\}\;\left(i=1,\;2,\;\cdots ,\;k\right)$$

where y(i) is the extreme lowest position and k denotes the sample number, $y_{a}$ represents the average value of extreme lowest position.

The introduction of the deviation position value would illustrate the different of average displacement for corresponding slider in the different rotation speeds, which shows the ability of accuracy maintenance for press systems. The smaller value explains that press systems has a better stability and a larger scope of processing technology, which is written as:

(2) $$\gamma_{2}=y_{a}^{j}-y_{a}^{i}\;\left(j=1,\;2,\;\cdots ,\;q\right)$$

where $y_{a}^{j}$ is the average value of extreme lowest position in the different rotation speeds, q denotes the order number of rotation speed and $y_{a}^{i}$ represents the ideal average value of extreme lowest position.

The mean square error can explain the stability of extreme lowest position in the same working condition, and the consistency of manufactured product accuracy is also reflected. The expression is displayed as:

(3) $$\gamma_{3}=\sqrt{\frac{1}{k}\sum_{i=1}^{k}{\left[y\left(i\right)-y_{a}\right]}^{2}}\;\left(i=1,\;2,\;\cdots ,\;k\right)$$

**Fig. 1:**
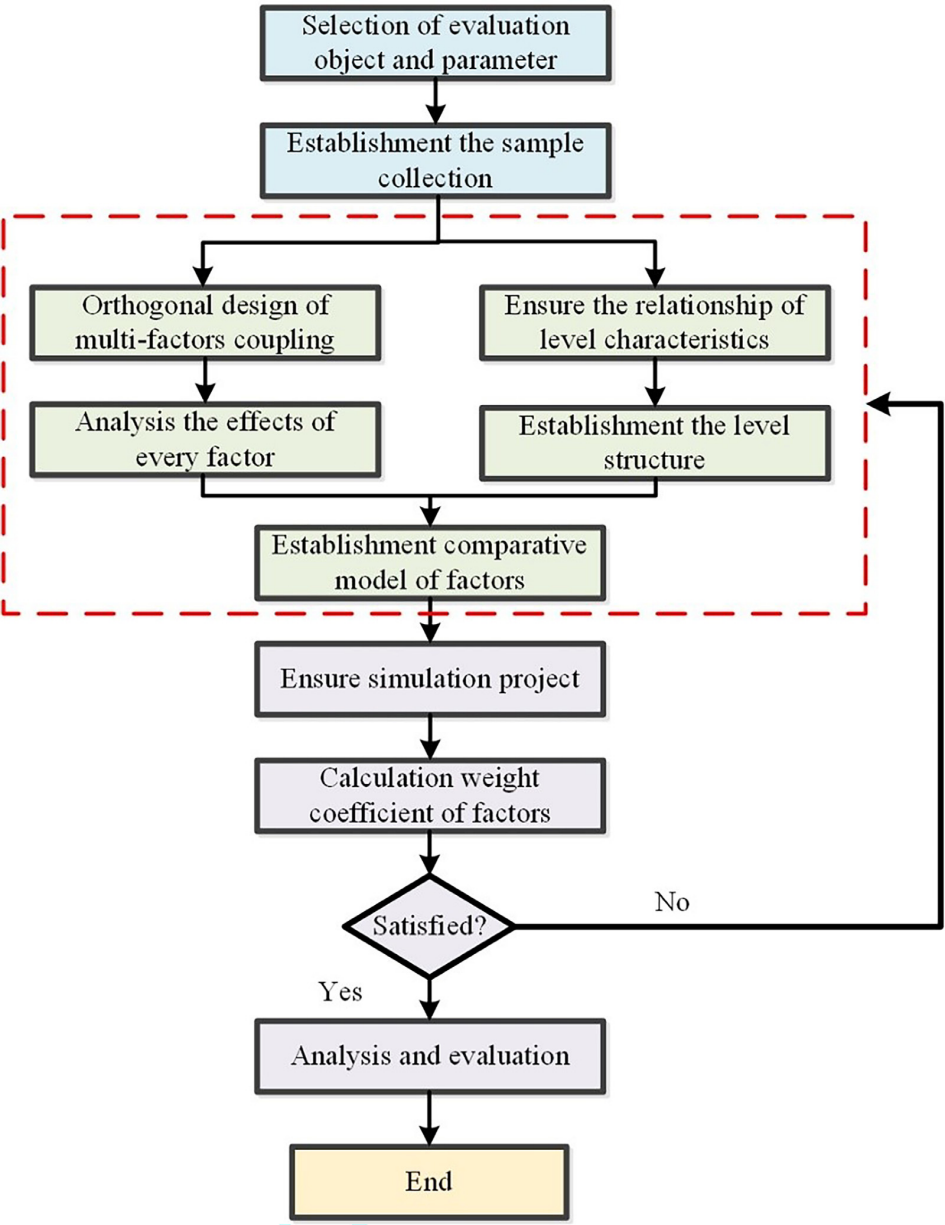
The flow chart of comprehensive evaluation systems.

The comprehensive evaluation systems should be established in the dynamic accuracy study, which can promote that the quantitative analysis is used to conduct the qualitative problem. The flow chart of comprehensive evaluation systems is shown in Fig. 1. Based on the requirement of stability evaluation, it is need to select the suitable object and parameter. Then, the sample of dynamic accuracy is collected, which is the extreme lowest position of slider. Moreover, the evaluation model of dynamic accuracy for press systems is established by the orthogonal design method. The coupled effects of multi-factors and level characteristics are analyzed and compared. The weight characteristics of factors is calculated and order number of factors is listed. In addition, the analysis and evaluation of dynamic accuracy for press systems are conducted. The dynamic accuracy is the main factor to evaluate the ability of press system, which should be selected to the evaluation. And, the mathematical statistics results are employed to analyze the dynamic behavior of press systems in the multi-dimensional factor. The dynamic response of press system is close related with the working condition and structure parameters, and the collection sample is defined. Moreover, the computation of dynamic accuracy evaluation can be simplified by the orthogonal design method. Then, the efficiency of computing and evaluation can be improved.

### Orthogonal design method

**Fig. 2:**
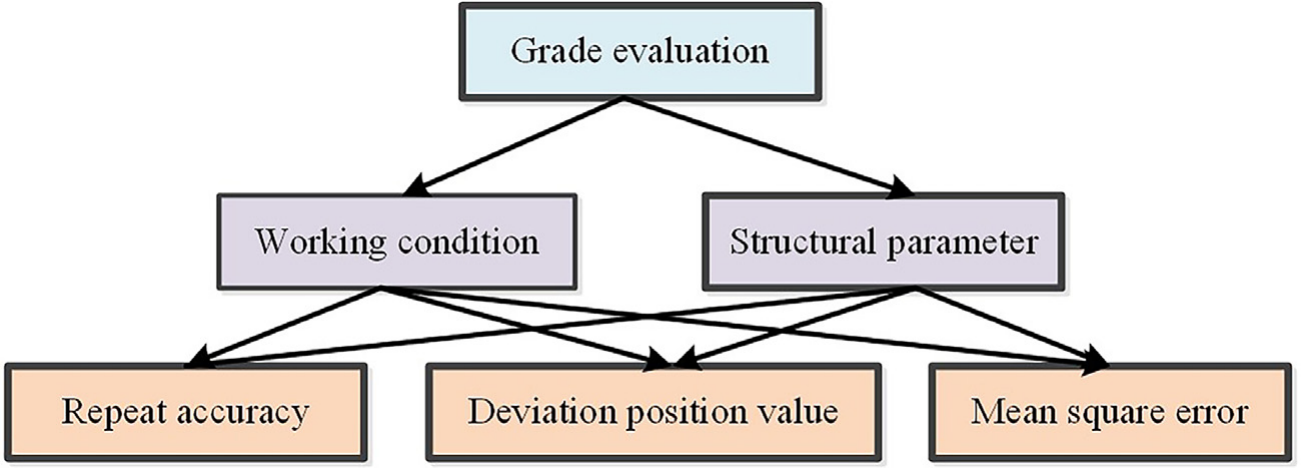
The level structure of dynamic accuracy.

Due to the effects of manufacturing quality on the dynamic accuracy are obvious, the working condition and structure parameters are selected to the influence factor. Meanwhile, the repeat accuracy, the deviation position value and the mean square error are chosen to the evaluation element. The level structure of dynamic accuracy is built, as shown in Fig. 2. Based on the orthogonal design method, the orthogonal table ($L_{n}\left(r^{m}\right)$) is established, which would consider the effect degree and interaction of factors. The orthogonal analysis table is given in Table 1. The difficult changed factor should be considered in the first order number. Then, the simulation project is conducted and the weight coefficient of factors is obtained. In addition, the analysis and evaluation of dynamic accuracy for press systems are performed, which would provide the reference for the design of production.

**Table 1:** Orthogonal analysis table.

Case number	$A_{i}$	$B_{i}$	$C_{i}$
1			
2			
⋯			
n			

### Case study

**Fig. 3:**
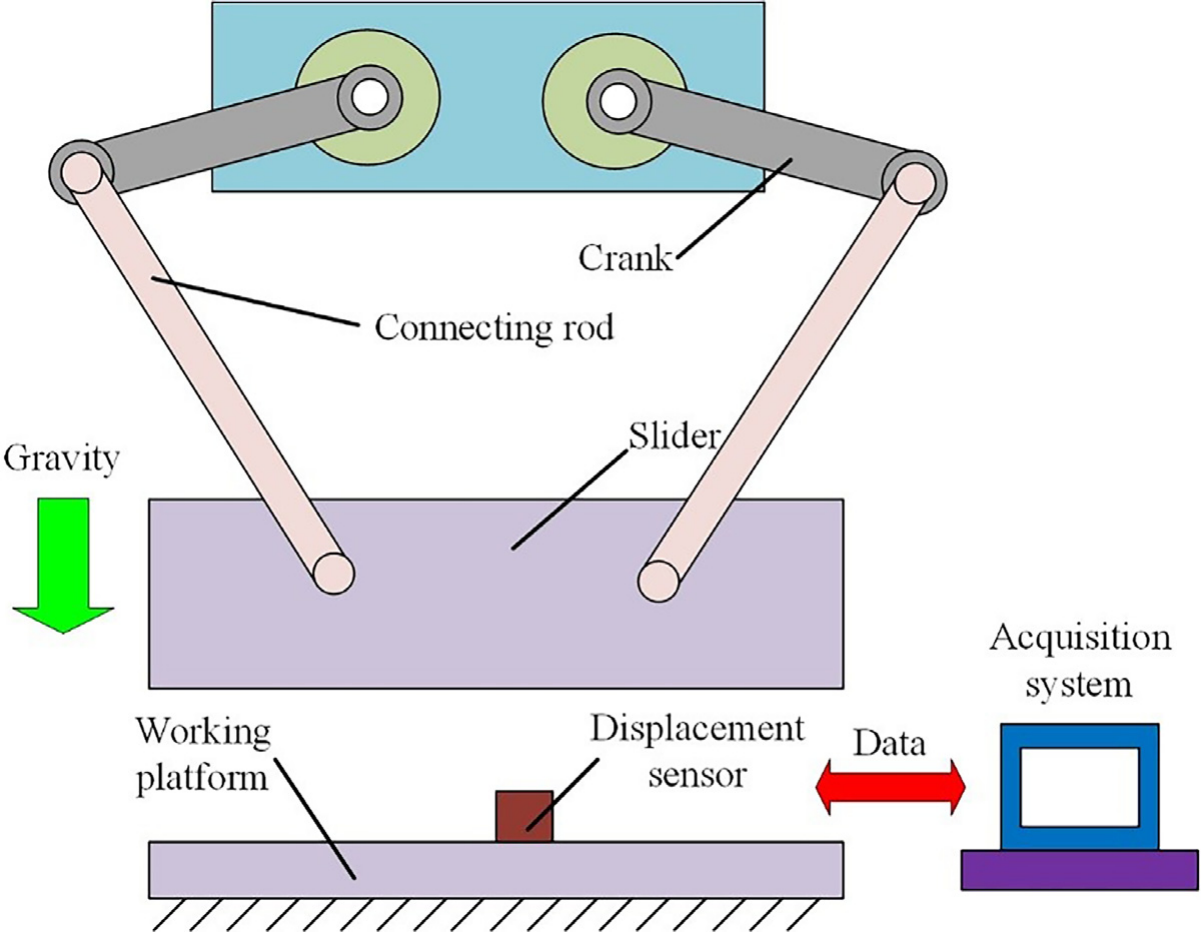
The schematic of transmission mechanism for press systems.

The case study could illustrate the application of proposed method, and the schematic of transmission mechanism for press systems is displayed in Fig. 3. The lengths of crank and connecting rod are 0.014 m and 0.35 m. The mass of crank, connecting rod and slider are 102 kg, 412 kg and 2700 kg, respectively. And then, the clearance is defined to the revolute joint, which is the connection of mechanical elements. With the driven of crank, the slider conducts the reciprocating motion. The working platform is fixed on the ground and the displacement sensor is installed on the working platform, which can record the value of extreme lowest position for slider. Then, the sample collection is obtained and stored in the acquisition systems. In addition, the analysis and evaluation of dynamic accuracy for press systems is conducted. Significantly, the calculation of dynamic accuracy evaluation can be conducted by computer. The Eqs (1), (2) and (3) are written by the M-file in Matlab [9]. Moreover, the analysis data can be obtained by experiment or simulation. And, he Runge-Kutta algorithm in Matlab can be used to solved the dynamic simulation of press system [3,6].

Due to the effects of dynamic accuracy for press systems is the complex problem, the selection of design parameter plays an important role to the study of stability. According to the feature of press systems, the rotation speed ($A_{i}$), clearance size ($B_{i}$), working stroke ($C_{i}$) and slider mass ($D_{i}$) are defined to the effect factors. Then, the repeat accuracy (RA), deviation position value (DPV) and mean square error (MSE) are chosen to the evaluation elements. In general, the number of case study is need to 4^3^ (64) times. Based on the orthogonal design method, the orthogonal analysis table is built ($L_{9}\left(3^{4}\right)$). The design parameters of effects factors are listed to $A_{i}$ (100 rpm, 200 rpm, 300 rpm), $B_{i}$ (0.01 mm, 0.02 mm, 0.03 mm), $C_{i}$ (0.012 m, 0.014 m, 0.016 m) and $D_{i}$ (2400 kg, 2700 kg, 3000 kg). The calculation of dynamic accuracy for press systems is given in Table 2, and the calculation time is only 9, which illustrates that the orthogonal design could simplify the case number and establish the relationship of multi-factors. It is clear seen that the multi-factors have different effects on the dynamic behavior of press systems. Although the case number is reduced by the orthogonal design, the relationship between design parameter and dynamic accuracy is not intuitive enough. Then, the weight coefficient (*k*_i_) is also calculated and the limit difference (LD) is obtained, which is shown in Table 3. According to the results, the repeat accuracy is more sensitive to the variation of rotation speed and clearance size. The increase of clearance size would enlarge the motion range of component and the nonlinear contact phenomenon would appear obviously, which is the main reason for the vibration of dynamic accuracy. Meanwhile, the rotation speed has an obvious effects on the deviation position value. The reason of this phenomenon can be explained that the growth of rotation speed increases the inertial force of slider during the motion. Compared with the traditional mechanism, the slider has a heavy weight and the connecting rod would be produced to elastic deformation, which causes the decline of deviation position value. The mean square error illustrates the effects of multi-factors on the manufacturing quality of production. It is clear that the rotation speed and working stroke are the important roles in the stability of dynamic accuracy.

**Table 2:** Calculation of dynamic accuracy for press systems.

Case number	$A_{i}$	$B_{i}$	$C_{i}$	$D_{i}$	RA (mm)	DPV (mm)	MSE (mm)
1	1	1	1	1	0.0093	0.0018	3.811 × 10^−6^
2	1	2	2	2	0.0325	0.0019	1.872 × 10^−5^
3	1	3	3	3	0.0543	0.0028	8.537 × 10^−6^
4	2	1	2	3	0.0148	0.0054	1.458 × 10^−4^
5	2	2	3	1	0.0278	0.0073	4.941 × 10^−6^
6	2	3	1	2	0.0652	0.0037	2.583 × 10^−5^
7	3	1	3	2	0.0099	0.0135	2.951 × 10^−5^
8	3	2	1	3	0.0239	0.0088	2.021 × 10^−4^
9	3	3	2	1	0.0448	0.0124	2.762 × 10^−4^

**Table 3:** Simulation results analysis.

		$A_{i}$	$B_{i}$	$C_{i}$	$D_{i}$
RA (mm)	k_1_	0.0961	0.0340	0.0984	0.0819
	k_2_	0.1078	0.0842	0.0921	0.1076
	k_3_	0.0786	0.1643	0.0920	0.0930
	LD	0.0292	0.1303	0.0064	0.0257
	Effects	*B*>*A*>D>C			
DVP (mm)	k_1_	0.0065	0.0207	0.0143	0.0215
	k_2_	0.0164	0.0180	0.0197	0.0191
	k_3_	0.0347	0.0189	0.0236	0.0170
	LD	0.0282	0.0027	0.0093	0.0045
	Effects	A>C>D>B			
MSE (× 10^−6^ mm)	k_1_	14.220	179.121	231.741	284.952
	k_2_	176.571	225.761	440.720	74.060
	k_3_	507.81	310.567	42.988	356.437
	LD	493.590	131.446	397.732	282.377
	Effects	A>C>D>B			

**Fig. 4:**
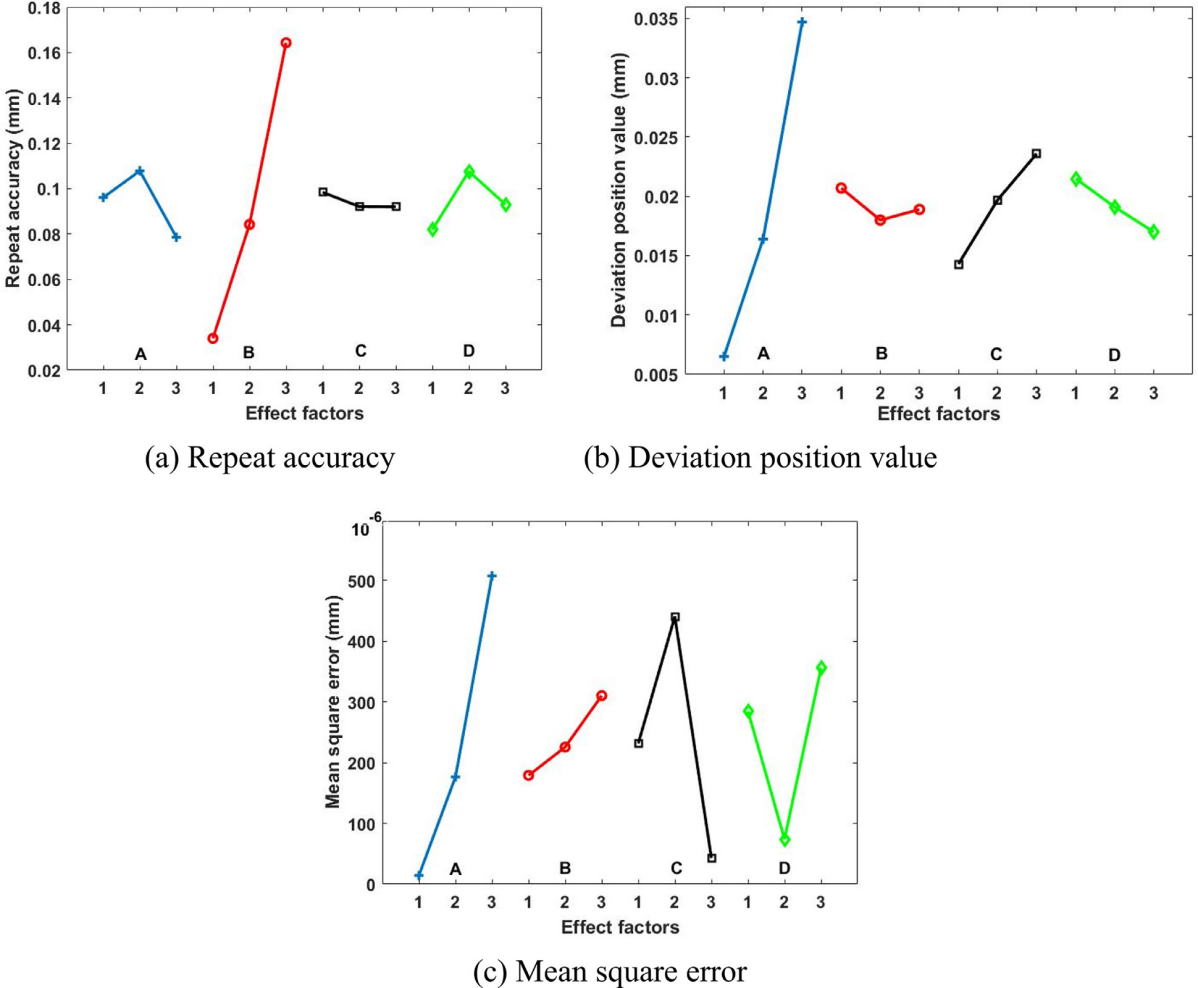
Effects characteristics of evaluation elements.

Fig. 4 represents the changed trend of multi-factors effects on the dynamic accuracy. When the clearance size is smaller, the lower rotation speed is not necessarily able to provide the better repeat accuracy and stability. However, the growth of rotation speed could cause the deviation of extreme lowest position. It is worth note that the order of multi-factors influence can be given by the evaluation and analysis results (A>C>*B*>*D*).

## Summary comments

The evaluation and analysis method of dynamic accuracy for press systems is represented in this study, which has the general application for the high-speed and ultra-precision mechanism. Based on the design object, the comprehensive evaluation systems of dynamic accuracy is established. Then, the evaluation elements (repeat accuracy, deviation position value and mean square error) could reveal the stability and reasonableness of evaluation systems. Moreover, the introduction of orthogonal design method simplifies the case number of multi-factors analysis. The case study represents the coupling influence relationship between design parameters and evaluation elements. In addition, the application of this evaluation and analysis method can be extended to the industrial engineering. For example, the motion trajectory evaluation of spacecraft and robot can be used to optimize the design of structure. Then, the matching relationship between dynamic behavior and structure parameters can be reflected in the precision transmission mechanism.

## Declaration of competing interest

The authors declare that they have no known competing financial interests or personal relationships that could have appeared to influence the work reported in this paper.

## Data Availability

No data was used for the research described in the article.
